# Veno-venous extracorporeal membrane oxygenation as a bridge in central airway obstruction: experience from a high-volume center

**DOI:** 10.1186/s13054-024-05219-0

**Published:** 2024-12-20

**Authors:** Xiao-xiu Luo, Jia-jia Li, Fu-xun Yang, Yu Lei, Fan Zeng, Yun-ping Lan, Chun Pan, Xiao-bo Huang, Rong-an Liu, Jing-chao Luo

**Affiliations:** Department of Critical Care Medicine, University of Electronic Science and Technology of China, Sichuan Academy of Medical Sciences and Sichuan Provincial People’s Hospital, Chengdu, 610072 China

**Keywords:** ECMO, Central airway obstruction, Minimal anticoagulation protocol, Cannulation strategy

## Abstract

**Background:**

Perioperative airway management and oxygenation maintenance during central airway obstruction (CAO) treatment pose great challenges. While veno-venous extracorporeal membrane oxygenation (V-V ECMO) shows promise as a bridge therapy, optimal implementation and management strategies remain lacking. We present our experience with V-V ECMO in CAO management from a high-volume center.

**Methods:**

We retrospectively analyzed 29 consecutive patients who received V-V ECMO support for CAO between 2015 and 2023. Patient demographics, clinical characteristics, ECMO cannulation and operation parameters, interventional procedures, complications, and outcomes were reviewed.

**Results:**

Among patients with median airway diameter of 4.5 mm (IQR 2–5 mm), etiologies included primary tumors (n = 17), metastases (n = 7), and post-intubation/tracheostomy stenosis (n = 5). Treatment comprised bronchoscopic interventions (n = 9) and surgical procedures (thoracic = 15, head/neck = 5). Using predominantly femoral-jugular cannulation (n = 27), we implemented a minimal anticoagulation protocol (catheter flush with 5U/mL heparin only). All patients survived through 6-month follow-up with minimal ECMO-related complications.

**Conclusion:**

The application of V-V ECMO with minimal anticoagulation demonstrates safety and efficacy as a bridging support in the therapeutic approach to CAO.

**Supplementary Information:**

The online version contains supplementary material available at 10.1186/s13054-024-05219-0.

## Introduction

Central airway obstruction (CAO) is characterized by a blockage of airflow in the trachea and main bronchus, presenting a life-threatening emergency [1]. CAO can result from both benign and malignant diseases, including local invasion and distant metastasis of lung, thyroid, and esophageal cancers; tracheal foreign body; tracheomalacia; tracheotomy or lung transplantation; anastomotic stenosis; and local obstruction caused by airway bleeding. Changes in the cross-sectional area of the airway may affect its Reynolds coefficient [2], resulting in various clinical manifestations. Acute inspiratory wheezing and respiratory distress are critical signs of CAO, necessitating timely intervention; otherwise, the risk of death is extremely high. However, ensuring patient safety during therapeutic interventions, particularly maintaining adequate oxygenation, remains a formidable challenge.

In recent years, veno-venous extracorporeal membrane oxygenation (V-V ECMO) has emerged as an increasingly adopted respiratory support strategy for CAO management. However, standardized protocols for its implementation in this specific clinical scenario remain to be established. Since the pioneering application of extracorporeal circulation for thyroid tumor-induced airway obstruction in 1999, refinements in this technology have facilitated successful perioperative management, with numerous subsequent reports documenting its efficacy in various airway obstructive conditions [3–7]. Despite these advances, concerns have arisen regarding complications such as hemorrhagic pleural effusion associated with systemic heparinization [8]. Early V-V ECMO intervention can prevent catastrophic hypoxemia during both the natural course and therapeutic management of airway obstruction; however, concerns exist regarding cost implications and potential overtreatment. Furthermore, the optimal anticoagulation protocol for short-term airway support remains controversial.

This report describes our nine-year institutional experience regarding V-V ECMO as a bridging support in the therapeutic management of CAO.

## Methods

### Patients

We reviewed patients who received V-V ECMO support for CAO treatment at our center between 2015 and 2023. The Ethics Committee of Sichuan Provincial People's Hospital approved this study (No. 2022-91), waiving the requirement for informed consent due to the retrospective, anonymized nature of data analysis. Patients who completed their therapeutic interventions for CAO were included in the final analysis.

### Data collection

The following variables were collected: demographics, underlying conditions, American Society of Anesthesiologists (ASA) physical status classification, CAO characteristics (position of obstruction, airway management status, narrowest airway diameter), V-V ECMO parameters (cannulation approach, anticoagulation protocol, support duration), surgical interventions, hospital length of stay, complications, and outcomes.

### Statistical analysis

Data analysis was performed using SPSS 20.0 (IBM Corporation, NY). Continuous variables were expressed as median (IQR) and compared using Wilcoxon's rank-sum test, while categorical variables were presented as frequencies (percentages) and compared using Fisher's exact test. Statistical significance was set at *p* < 0.05.

## Results

### Participants

Of the 31 patients initially screened, two patients abandoned further endobronchial treatment and were excluded: one due to uncontrollable tumor hemorrhage during bronchoscopy, and another due to fatal ECMO complications (acute cardiac tamponade during cannulation necessitating emergency sternotomy, followed by withdrawal of life support and death one week after ECMO initiation). Twenty-nine patients were ultimately included in the final analysis. (Table 1 and Table S2). The etiologies of CAO included primary tumors (n = 17), metastatic tumors (n = 7), and post-intubation/tracheostomy stenosis (n = 5). While baseline characteristics were comparable across groups, patients with iatrogenic stenosis were significantly younger (median age 27[IQR 19–52], *P* = 0.04). All patients presented severe airway obstruction (median narrowest diameter 4.5 mm [IQR 2–5]), with no significant differences between etiologies (*P* = 0.268). (Table 1).

**Table 1:** Basic Characteristics of patients

The Cause of obstruction	Total	Primary tumor	Tumor metastasis	Artificial airway	*P* value
Number	29	17	7	5	
Age	52 (46–69)	57 (43–68)	57 (51–71)	27 (19–52)	0.040
Gender (male, %)	18 (62.1)	11 (64.7)	4 (57.1)	3 (60)	0.939
ASA Classification					0.280
II	1 (3.4)	0	1 (14.2)	0	
III	15 (51.7)	11 (64.7)	1 (14.2)	3 (60)	
IV	11 (37.9)	6 (35.3)	3 (42.8)	2 (40)	
V	2 (6.8)	0	2 (28.6)	0	
Position of obstruction					0.830
I	12 (41.4)	6 (35.3)	3 (42.8)	3 (60)	
II	8 (27.6)	5 (29.4)	2 (28.6)	1 (20)	
III	2 (6.8)	1 (5.9)	1 (14.2)	0	
IV	5 (17.2)	3 (17.6)	1 (14.2)	1 (20)	
V	2 (6.8)	2 (11.8)	0	0	
Narrowest Airway Diameter (mm)	4.5 (2–5)	3 (2–4)	3 (2–5)	5 (2.5–5.5)	0.268
Artificial Airway (Yes, %)	16 (55.1%)	9 (52.9%)	3 (42.9%)	4 (80%)	0.438

Position of obstruction: I = Upper third of the trachea; II = Middle third of the trachea; III = Lower third of the trachea; IV = Right main bronchus; V = Left main bronchus

ASA Classification, American Society of Anesthesiologists Physical Status Classification System

### ECMO cannulation strategy

**Fig. 1:**
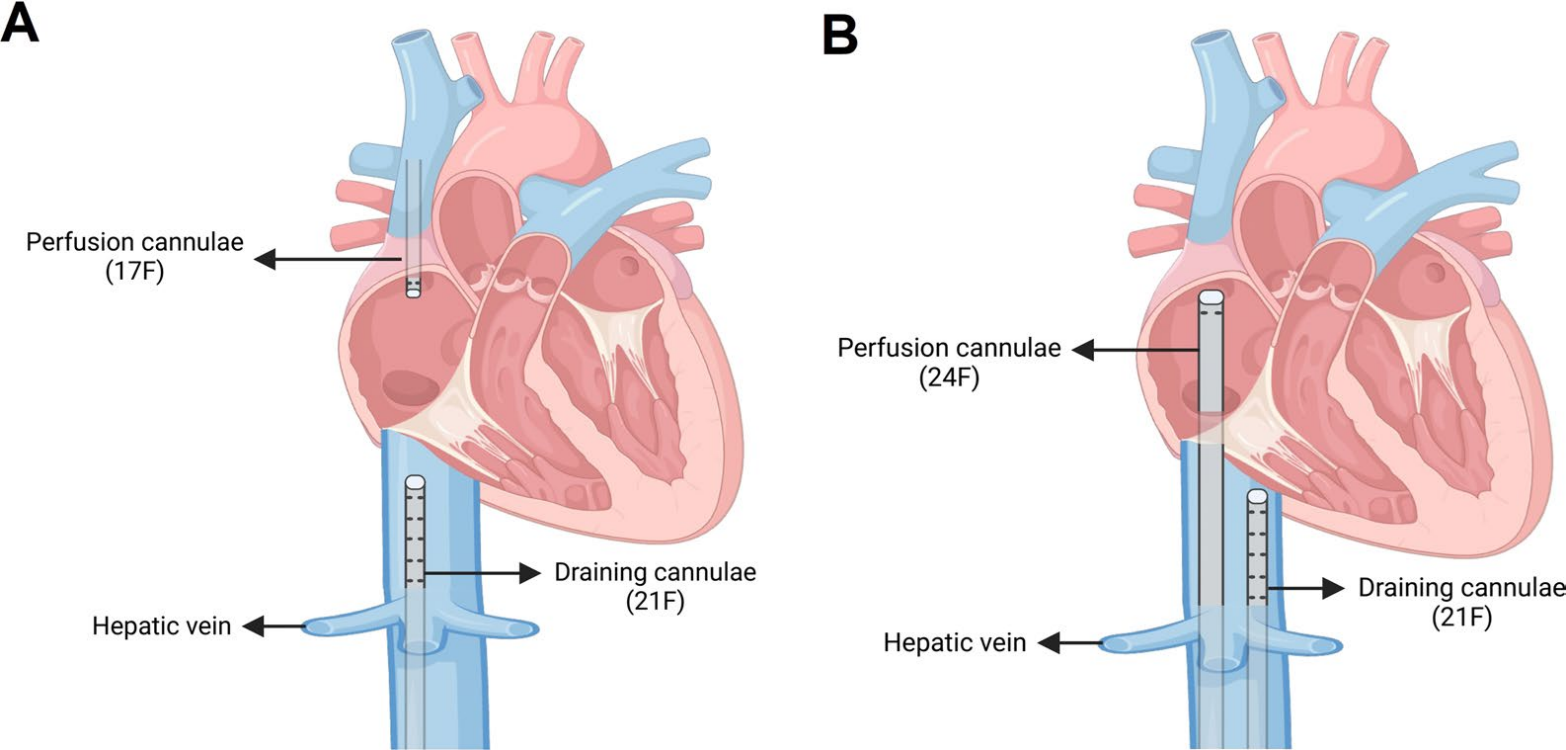
ECMO Cannulation Modes. Panel **A**: femoral to internal jugular configuration (primary strategy) applied in the majority of cases: 21F catheter inserted via femoral vein to the hepatic vein opening of IVC, and 17F catheter inserted via internal jugular vein to a depth of 13–15 cm; Panel **B**: femoral to veno-femoral configuration (alternative strategy for neck surgery): 21F catheter inserted via femoral vein to the hepatic vein opening of IVC, and 24F catheter inserted via contralateral femoral vein to the SVC-RA junction. All cannulations were performed under ultrasound guidance. IVC, inferior vena cava; SVC, superior vena cava; RA, right atrium; VV-ECMO, veno-venous extracorporeal membrane oxygenation

Given that all patients had CAO, cannulation was performed under local anesthesia while maintaining spontaneous breathing, with minimal dexmedetomidine sedation administered only for one agitated patient; all procedures were completed without requiring general anesthesia. The majority of patients (n = 27) underwent femoral-jugular cannulation using 21F drainage and 17F return cannulas, with the femoral catheter positioned at the hepatic vein-IVC junction and jugular return catheter advanced 13–15 cm (Fig. 1A). Femoral-femoral configuration (21F drainage, 24F return) was utilized in two patients where neck access was contraindicated or patient positioning (reverse Trendelenberg) precluded jugular cannulation, with the return catheter positioned at the SVC-RA junction (Fig. 1B). All procedures were ultrasound-guided. Avalon Elite® cannulas were not used due to cost considerations and potential surgical interference.

### Minimal anticoagulation protocol

None of the 29 patients received a heparin loading dose during cannulation. After successful catheterization, catheters were flushed with dilute heparin solution (5U/mL, total dose 500U per patient). ECMO flow was consistently maintained above 2L/min. Due to the risk of airway bleeding, systemic anticoagulation was initially avoided, and daily ultrasound monitoring was performed for potential thrombotic events. Only two patients with a history of atrial fibrillation were subsequently initiated on systemic heparinization once their postoperative bleeding risk had diminished. No ECMO-related bleeding or thrombotic complications occurred, and post-operative hemoglobin and platelet counts remained stable (Table S1).

### Treatment modalities and outcome

**Fig. 2:**
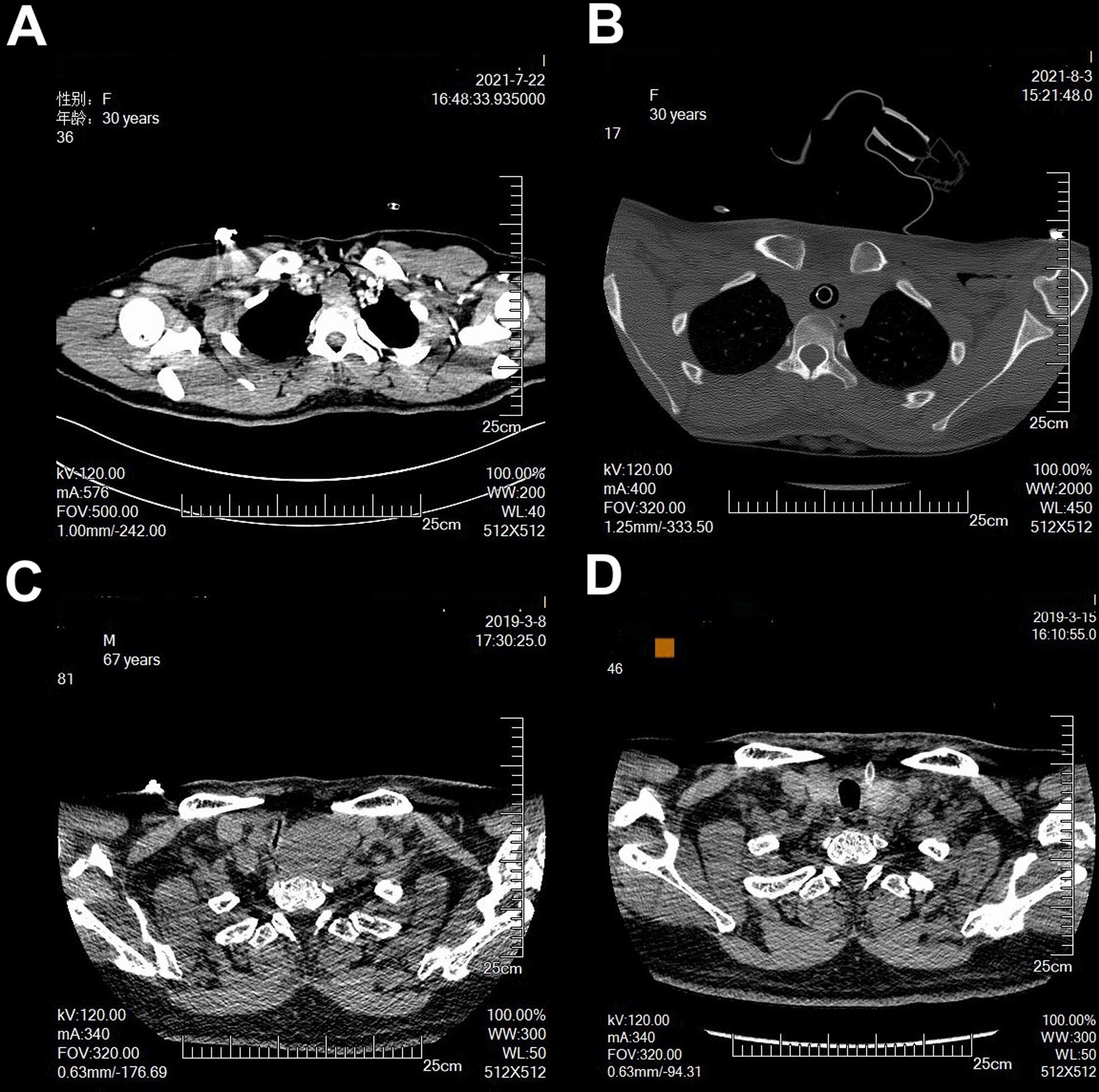
Representative pre- and post-intervention images. Panel **A**: Complex Tracheal Tumor with Acute Respiratory Compromise; Panel **B**: Successful Reconstruction after Tumor Resection; Panel **C**: Life-threatening Thyroid Mass with Severe Hypercapnia; Panel **D**: Complete Resolution after Thyroidectomy

Sixteen patients required artificial airways (12 intubations, 4 tracheostomies) during airway procedures, whereas the remaining patients were managed with sedation and analgesia alone. Nine patients underwent bronchoscopic interventions (ablation, cryotherapy, balloon dilation, stenting). Twenty underwent surgery: 15 thoracic procedures (tracheal tumor resection and reconstruction) and 5 head/neck operations (thyroid-related compression) (Fig. 2). ICU and hospital stays were comparable across intervention groups, with all patients surviving through 6-month follow-up (Table 2).

**Table 2:** Outcomes of patients

The Cause of obstruction	Primary tumor	Tumor metastasis	Artificial airway	*P* value
Number	17	7	5	
Surgical type				0.030
Otorhinolaryngological surgery	4 (23.5%)	0 (0%)	1 (20%)	
Thoracic Surgery	9 (52.9%)	2 (28.6%)	4 (80%)	
Interventional bronchoscopy	4 (23.5%)	5 (71.4%)	0 (0%)	
ICU stay duration	3 (2–8)	7 (3–14)	6 (3–9)	0.321
Hospital stay duration	18 (13–20)	13 (11–28)	13 (9.5–17.5)	0.511
28-Day mortality rate	0 (0%)	0 (0%)	0 (0%)	1.000
6-Month mortality rate	0 (0%)	0 (0%)	0 (0%)	1.000

ICU, Intensive Care Unit

### Complications

During emergency ECMO placement in a preoperative patient with severe airway obstruction, conventional airway management proved impossible, necessitating awake cannulation. During the procedure, the patient developed pneumothorax, likely due to forced positioning and vigorous spontaneous respiratory efforts. Immediate chest tube placement was performed concurrent with ECMO cannulation, and the patient was successfully stabilized.

## Discussion

This study represents the largest systematic investigation evaluating the efficacy and safety of V-V ECMO support in CAO patients, addressing a critical knowledge gap in this unique patient population.

Traditional management of CAO-induced hypoxemia predominantly relies on high-frequency ventilation, characterized by sub-dead space tidal volumes and extremely high respiratory rates. However, this approach carries substantial risks: shortened breathing cycles, combined with intrinsic PEEP and dynamic hyperinflation, can precipitate barotrauma and hemodynamic instability [9]. While VV-ECMO has emerged as a protective respiratory support strategy for CAO management with demonstrated safety and efficacy [10–12], criteria for prophylactic ECMO implementation remain unclear. Clinical evidence suggests that exertional dyspnea typically manifests when tracheal lumen decreases to < 8 mm, with wheezing becoming prominent at < 5 mm [13]. Beyond anatomical considerations, clinical presentation is influenced by spontaneous breathing intensity, airflow patterns, and pressure–volume loop dynamics [14]. Kim et al. [10] specifically advocated for ECMO support when bronchoscopic or CT findings demonstrate tracheal stenosis < 5 mm.

While intraoperative ECMO implementation remains an option, this reactive approach carries significant risks. In our cohort, most patients presented with airway diameters < 5 mm and enhanced respiratory drive, with severe dyspnea often precluding supine positioning—complicating catheterization procedures. Emergency ECMO placement not only increases procedural risks but also proves ineffective during respiratory arrest due to insufficient rapid oxygenation. Therefore, we propose ECMO as a preemptive rather than rescue measure, particularly for patients with airway diameters < 5 mm exhibiting three depressions sign. For thyroid tumors specifically, while some patients tolerate intubation under high-frequency oscillatory ventilation, those with tumors involving the trachea or anterior mediastinum, airway diameters < 5 mm, and firm, poorly mobile masses should be considered for prophylactic VV-ECMO due to risks of ventilation difficulties and airway complications from severe stenosis and upper airway deformities [15].

Cannulation strategy is a critical consideration in CAO patients, where we propose two approaches: femoral-internal jugular and femoral-femoral cannulation. Our experience supports bilateral femoral vein cannulation as the preferred method, which is particularly advantageous for neck procedures and patients with positioning restrictions (i.e., reverse Trendelenberg). While the main challenge with femoral-femoral approach is inadequate oxygenation due to ECMO recirculation, we address this by positioning the drainage catheter at the inferior vena cava-right atrial junction and advancing the return catheter deeper near the tricuspid valve to minimize recirculation. This approach proves especially valuable for patients requiring neck surgery or those with large cervical masses where jugular access is challenging or contraindicated. We strongly advocate ultrasound guidance for both vessel puncture and catheter tip positioning during the cannulation procedure.

Historically, ECMO circuits required intensive anticoagulation. However, technological advances, particularly in membrane materials and circuit coatings, have enabled minimal anticoagulation protocols. Studies have demonstrated successful ECMO support without heparin anticoagulation in high bleeding risk scenarios, including craniocerebral trauma [16] and pulmonary hemorrhage [17], showing no increased thrombotic complications and favorable survival outcomes. Lang et al. reported successful ECMO implementation during open-chest surgery using only a single pre-intubation dose of heparin sodium (3000–5000 IU) to minimize intraoperative bleeding [18]. Similarly, a retrospective analysis showed that administering only a 3000 IU heparin loading dose in high-bleeding-risk patients, without subsequent anticoagulation, neither increased adverse events nor affected mortality [19]. In our protocol, we further reduced the initial heparin dose to < 1000 U and maintained ECMO support without additional anticoagulation, observing no thrombotic complications. While this minimal anticoagulation approach enhances the safety of CAO procedures and facilitates postoperative recovery, we recommend continuous monitoring and assessment of thrombotic risk, with anticoagulation initiated when clinically indicated.

## Conclusion

V-V ECMO serves as a safe and effective bridge therapy for CAO management, particularly in patients with severe airway stenosis (< 5 mm). Our experience demonstrates that early implementation with minimal anticoagulation protocols facilitates successful airway interventions while maintaining favorable outcomes.

## Supplementary Information

Additional file1 (PDF 146 KB)

## Data Availability

No datasets were generated or analysed during the current study.
