# Supplementary material for: Veno-venous extracorporeal membrane oxygenation as a bridge in central airway obstruction: experience from a high-volume center
Source: Crit Care. 2024 Dec 20;28:426. doi: 10.1186/s13054-024-05219-0 (PMC11660460; doi:10.1186/s13054-024-05219-0)
Supplement: Supplementary file 1 — Additional file1 (PDF 146 KB) [file 13054_2024_5219_MOESM1_ESM.pdf]

Table S1. ECMO related parameters

ECMO-Related Indicators	Primary Tumor	Tumor Metastasis	Artificial Airway	P value
Number	17	7	5	
ECMO Flow (L/min)	3(3-5)	3.5(2-4)	3.3(3.1-3.9)	0.298
ECMO Operating Time	26(18-51)	19(12-28)	34(15-61)	0.494
Anticoagulated (Yes, %)	2(11.8%)	0(0%)	0(0%)	0.481
ECMO-Related Complications (Thrombosis/Bleeding)	0(0%)	0(0%)	0(0%)	1.000
Preoperative HGB	134(125-145)	120(98-130)	114(112-128)	0.037
Postoperative HGB	110(98-121)	91(88-123)	114(103-119)	0.547
Preoperative PLT	201(150-256)	205(165-247)	238(169-302)	0.518
Postoperative PLT	150(128-212)	173(130-223)	170(132-223)	0.750
Preoperative APTT	27.3(25.6-29.3)	26.8(26.1-30.6)	28.2(25.7-33.7)	0.629
Postoperative APTT	29.5(24.7-32.7)	35.2(32.3-36.9)	32.3(27.3-37.3)	0.171
Blood Transfusion	0(0%)	2(28.6%)	0(0%)	0.038

Table S1 Case series of VV ECMO in airway obstruction

Case	Age	Gender	CAO Causes	Intervention	Complications	Anticoagulation	Run time (h)	Flow(L/min)	Artificial airway	Tumor size	Position of obstruction	Diameter of the narrowest part of the airway	Survival (6m)
1	66	1	1	3	0	0	20	4	0	1.0cm*1.1cm	IV，V	2mm	1
2	39	1	1	2	0	0	26	2.7	1	1.9*1.8*1.9cm	II	2mm	1
3	33	1	1	3	0	0	5	2.4	1	1.5*1cm	I	5mm	1
4	60	1	1	3	0	0	120	3	0	NA	IV	2mm	1
5	48	1	1	2	0	0	11	3	1	2.1*2.7cm	II	2mm	1
6	64	1	1	2	0	1	48	3.5	1	NA	IV	2mm	1
7	80	0	1	2	0	0	21	3.5	0	1.5*1.0cm	I	3mm	1
8	48	1	1	2	0	0	26	3	0	3.5cm	I	2mm	1
9	71	1	1	1	0	0	73	3	1	5.8cm×3.5cm	I	4.4mm	1
10	32	0	1	2	0	0	48	3	0	1.5cm	III	5mm	1
11	57	1	1	3	0	0	16	3	0	15*25*23mm	I	3mm	1
12	46	1	1	2	0	0	24	3.2	0	3.6*3.2*3.3cm	I	2mm	1
13	31	0	1	2	1	0	69	3	2	2*1.5*1.5cm	II	1mm	1
14	69	0	1	1	0	1	54	2	2	NA	NA	3mm	1
15	59	0	1	1	0	0	31	3	1	NA	NA	4．5mm	1
16	52	0	1	2	0	0	21	3.5	0	18*10*11mm	II	3.8mm	1
17	51	1	2	3	0	0	19	1.65	1	1.8*2.5cm	II	5mm	1
18	60	1	2	3	0	0	20	3.5	0	1.5cm	II	5mm	1
19	52	1	2	3	0	0	168	2	0	NA	I	6mm	1
20	75	0	2	3	0	0	28	3.5	0	5.5*6.8cm	I	1mm	1
21	46	0	2	2	0	0	18	4	1	1.8*2.2cm	III	2mm	1
22	57	1	2	2	0	0	12	4.3	0	1.2cm	I	3mm	1
23	71	0	2	3	0	0	5	3	1	NA	IV、V	3mm	1

24	17	1	3	1	0	0	5	3.3	1	NA	IV、V	4mm	1
25	27	1	3	2	0	0	24	4.4	0	NA	II	5mm	1
26	21	0	3	2	0	0	6	3.4	1	NA	I	1mm	1
27	48	1	3	2	0	0	44	3.2	1	NA	I	5mm	1
28	55	0	3	2	0	0	34	3	2	NA	I	6mm	1
29	69	1	1	1	0	0	78	3	2	NA	II	4mm	1

CAO causes: Primary airway tumors =1、Metastatic airway tumors=2、Artificial airway causes benign airway hyperplasia =3;

Intervention approach: Otorhinolaryngological surgery = 1; thoracic surgery = 2, interventional surgery = 3;

Artificial airway: 0 = No artificial airway=0; Endotracheal intubation=1; Tracheostomy=2.
